# 1506. Prevalence of Histoplasma Antigenuria among an outpatient Advanced HIV cohort in Kampala,Uganda

**DOI:** 10.1093/ofid/ofad500.1341

**Published:** 2023-11-27

**Authors:** Preethiya Sekar, Elizabeth Nalintya, Richard Kwizera, Claudine Mukashyaka, Godfrey Niyonzima, Olive Loryndah Namakula, Patricia Nerima, Ann Fieberg, Biyue Dai, Nathan C Bahr, Jayne Ellis, David Meya, David R Boulware, Radha Rajasingham

**Affiliations:** University of Minnesota, Minneapolis, Minnesota; Infectious Diseases Institute, Makerere University college of Health Sciences, Kampala, Kampala, Uganda; Infectious Diseases Institute, college of Health Sciences, Makerere University, Kampala, Kampala, Uganda; Infectious Diseases Institute, Kampala, Kampala, Uganda; Infectious Disease Institute, Kampala, Kampala, Uganda; Infectious Disease Institute, Kampala, Kampala, Uganda; Infectious Disease Institute, Kampala, Kampala, Uganda; University of Minnesota, Minneapolis, Minnesota; University of Minnesota, Minneapolis, Minnesota; University of Kansas, Kansas City, Kansas; Infectious Diseases Institute, Makerere University, Kampala, Kampala, Uganda; Infectious Diseases Institute, Makerere University, Kampala, Kampala, Uganda; University of Minnesota, Minneapolis, Minnesota; University of Minnesota, Minneapolis, Minnesota

## Abstract

**Background:**

Histoplasmosis is a major cause of mortality in persons with advanced HIV disease (CD4< 200 cells/mcL) and areas of endemicity are evolving. Presenting symptoms of histoplasmosis may overlap with that of tuberculosis (TB). The true burden of histoplasmosis remains unknown in persons with advanced HIV disease, in part due to poor diagnostic capacity. We sought to evaluate the prevalence of *Histoplasma* antigenuria among outpatients with advanced HIV disease in Kampala, Uganda.

**Figure:**
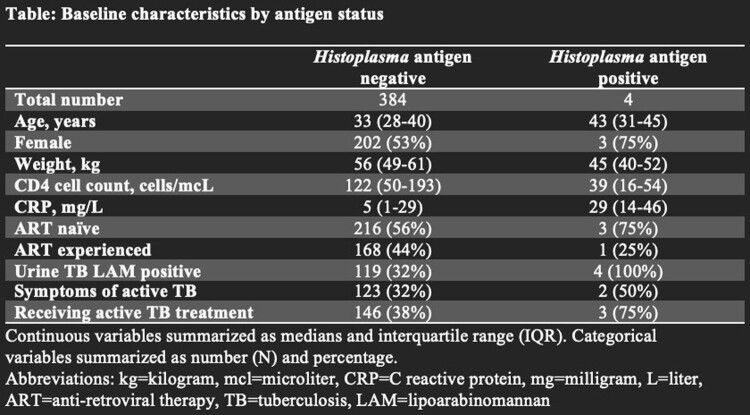

**Methods:**

This prospective cohort study of outpatients with advanced HIV in Kampala, Uganda was nested within the ongoing ENCORE trial evaluating preemptive therapy and prophylaxis among outpatients with advanced HIV disease. Urine samples were obtained from participants at the time of enrollment and a *Histoplasma* galactomannan enzyme immunoassay (EIA) (Immy, Norman OK) was run on urine per manufacturer’s instructions. We obtained baseline characteristics and laboratory values, a statistical comparison by antigen status was not done due to a low number of positives. We calculated the prevalence of histoplasmosis in our cohort using EIA results. We obtained information on symptoms, TB diagnostics, and TB treatment for those with *Histoplasma* antigenuria.

**Results:**

We tested 388 urine samples among participants with advanced HIV disease. Four samples were positive for *Histoplasma* antigen (1%). Baseline characteristics of participants are summarized in Table 1. *Histoplasma* antigen prevalence among participants with CD4< 100 cells/mcL was 2.5% (4/158). Of those that tested positive for histoplasmosis, the median CD4 count was 39 cells/mcL (interquartile range (IQR): 16–54), and median CRP was elevated at 29 (IQR 14-46). All four participants with *Histoplasma* antigenuria had a positive TB urine lipoarabinomannan (LAM, AlereLAM, Abbott, Palatine, IL, USA), though only two of four participants reported symptoms of cough, weight loss, and/or physical weakness. Three of four participants with positive *Histoplasma* antigen were started on anti-TB treatment. Patient symptoms resolved with no intervention.

**Conclusion:**

Among outpatients with severe advanced HIV disease with CD4< 100 cells/mcL in Kampala, Uganda *Histoplasma* antigen prevalence was 2.5%.

**Disclosures:**

**All Authors**: No reported disclosures

